# Significance of tumor heterogeneity of p-Smad2 and c-Met in HER2-positive gastric carcinoma with lymph node metastasis

**DOI:** 10.1186/s12885-022-09681-3

**Published:** 2022-06-01

**Authors:** Gen Tsujio, Koji Maruo, Yurie Yamamoto, Tomohiro Sera, Atsushi Sugimoto, Hiroaki Kasashima, Yuichiro Miki, Mami Yoshii, Tatsuro Tamura, Takahiro Toyokawa, Hiroaki Tanaka, Kazuya Muguruma, Masaichi Ohira, Masakazu Yashiro

**Affiliations:** 1 Department of Gastroenterological Surgery, Osaka Metropolitan University Graduate School of Medicine, 1-4-3 Asahimachi, Abeno-ku, Osaka, 545-8585 Japan; 2 Molecular Oncology and Therapeutics, Osaka Metropolitan University Graduate School of Medicine, 1-4-3 Asahimachi, Abeno-ku, Osaka, 545-8585 Japan; 3 Cancer Center for Translational Research, Osaka Metropolitan University Graduate School of Medicine, 1-4-3 Asahimachi, Abeno-ku, Osaka, 545-8585 Japan

**Keywords:** Gastric cancer, Intra-tumoral heterogeneity, Clustering analysis

## Abstract

**Background:**

Tumor heterogeneity has frequently been observed in gastric cancer (GC), but the correlation between patients’ clinico-pathologic features and the tumoral heterogeneity of GC-associated molecules is unclear. We investigated the correlation between lymph node metastasis and the intra-tumoral heterogeneity of driver molecules in GC.

**Materials and methods:**

We retrospectively analyzed the cases of 504 patients who underwent a gastrectomy at the Department of Gastroenterological Surgery, Osaka Metropolitan University and 389 cases drawn from The Cancer Genome Atlas (TCGA) data. We performed a clustering analysis based on eight cancer-associated molecules including HER2, c-Met, and p-Smad2 using the protein expression revealed by our immunohistochemical study of the patients’ and TCGA cases. We determined the correlations between HER2 expression and the other molecules based on the degree of lymph node metastasis.

**Results:**

Immunohistochemical staining data showed that a 43 of the 504 patients with GC (8.5%) were HER2-positive. In the HER2-positive cases, the expressions of c-Met and p-Smad2 were increased in accord with the lymph-node metastatic level. The overall survival of the HER2-positive GC patients with both p-Smad2 and c-Met expression was significantly (*p* = 0.030) poorer than that of the patients with p-Smad2-negative and/or c-Met-negative expression. The results of the TCGA data analysis revealed that 58 of the 389 GC cases (14.9%) were *ERBB2*-positive. *MET* expression was more frequent in the N1 metastasis group than the N0 group. In the high lymph-node metastasis (N2 and N3) group, *SMAD2* expression was more frequent, as was *ERBB2* and *MET* expression.

**Conclusion:**

p-Smad2 and c-Met signaling might play important roles in lymph node metastasis in HER2-positive GC.

**Supplementary Information:**

The online version contains supplementary material available at 10.1186/s12885-022-09681-3.

## Background

Tumor heterogeneity of cancer cells is frequently present in various types of cancer and has been reported to be associated with therapeutic issues including chemo-resistance and metastasis [1–5]. Tumor heterogeneity has been observed both between tumors as inter-tumoral heterogeneity and within tumors as intra-tumor heterogeneity. The intra-tumoral heterogeneity of cancer-associated molecules might play an important role in metastasis [6–9]. However, the correlation between intra-tumoral heterogeneity patterns and the process of metastasis has not been determined. A clarification of intra-tumoral heterogeneity patterns may be useful, as this may help explain the manner of metastasis and contribute to the selection of therapeutic targets.

We previously demonstrated the clinicopathological significance of several cancer-associated molecules including fibroblast growth factor receptor 2 (FGFR2), transforming growth factor-beta 1 (TGFβ1), C-X-C chemokine receptor 2 (CXCR2), CXCR4, and phospho-Smad2 expressed at primary gastric tumors [10–14]. HER2 and c-Met are also known as driver proteins of gastric cancer. The clarification of the correlation between the tumoral heterogeneity of these molecules and lymph node metastasis may contribute to the development of new treatment strategies.

Above all, HER2 has been widely accepted as an underlying treatment biomarker of gastric cancer, and anti-HER2 therapy with the monoclonal antibody trastuzumab is recommended as the first-line therapy for patients with HER2-positive advanced or metastatic gastric cancer. In contrast to breast cancer, significant intra-tumoral heterogeneity of HER2 expression was observed in gastric cancer, and in 4.8%–75.4% of HER2-positive gastric cancer case [15–17]. The HER2 expression’s intra-tumoral heterogeneity is related to the development of chemotherapy resistance, and it has become a serious clinical problem. An identification of the proteins / genes related to HER2 / *ERBB2* expression could contribute to new molecular target therapies for HER2-positive gastric cancer.

We thus conducted the present study to (1) examine the correlation between the molecular intra-tumoral heterogeneity and HER2 / *ERBB2* expression and lymph node metastasis in gastric tumors in order to clarify the mechanism(s) responsible for lymph node metastasis, and (2) investigate potential therapeutic targets against lymph node metastasis.

## Materials and methods

### Patients

A total of 504 gastric cancer patients who underwent gastrectomy at the department of Gastroenterological Surgery Osaka Metropolitan University between 2001 and 2006 was enrolled. Also, a total 389 cases of The Cancer Genome Atlas (TCGA) data were added in this study. The pathologic diagnoses and classifications were made according to the UICC TNM classification of malignant tumors.

### Immunohistochemical staining

The immunohistochemical determination of the tumor-associated molecules expression in gastric tumors was performed as follows. Bond Oracle™ HER2 IHC System (Leica Biosystems, Newcastle Upon Tyne, UK) was used for HER2 staining, according to the manufacturer’s instructions. HER2 expression were considered positive when intensity scores were ≥ 2 and proportion score were 10%. Immunohistochemical staining for CXCR2 (1:50; R&D Systems, Minneapolis, MN, USA), CXCR4 (1:100; Abcam, Cambridge, MA, USA), and FGFR2IIIb (1:333; Cell Signaling, Danvers, MA, USA) was performed as previously reported [10, 12, 13]. Immunohistochemical staining for c-Met (1:200; Santa Cruz Biotechnology, Dallas, TX, USA), IGF1R (Insulin-like growth factor 1 receptor) (1:500; Abcam, Cambridge, MA, USA), p-smad2 (1:2000; Chemicon International, Temecula, CA, USA), and TGFβ1 (1:100; Lab vision, Fremont, CA, USA) was performed as follows. Shortly, we performed deparaffinization and slides were heated. After blocking endogenous peroxidase activity, the sample were incubated with each antibody for 1 h at room temperature. The sample were incubated with biotinylated second antibody. The samples were treated with streptavidin-peroxidase reagent, and counterstaining with Mayer’s hematoxylin. The expression level was analyzed by both intensity of staining and percentage of stained cancer cells at the invading tumor front. Evaluation was made by two double-blinded independent observers who were unaware of clinical data and outcome. When a different evaluation between two independent observers was found, the evaluation was rechecked and discussed. The p-smad2 and c-Met expression were considered positive when intensity cores were ≥ 2 and proportion score were ≥ 40%.

### Clustering analysis of protein and gene expressions

The clustering analysis based on protein expression and gene expression of 8 molecules, including HER2 (*ERBB2*), c-Met (*MET*), CXCR2 (*CXCR2*), CXCR4 (*CXCR4*), FGFR2IIIb (*FGFR2*), IGF1R (*IGF1R*), TGFβ1 (*TGFB1*), p-smad2 (*SMAD2*) were conducted. The correlations among these molecules were analyzed according to the degree of lymph node metastasis. The mRNA expression Z-score of genes (RNA-Seq V2 RSEM normalized, RNA-Seq data) were obtained from 389 cases of TCGA stomach adenocarcinoma dataset (PanCancer Atlas) through cBioPortal [18]. Z-scores were used for clustering analysis.

### Protein expression for clustering analysis

Protein expression was evaluated with the intensity of staining and percentage of stained tumor cells. Intensity was scored 0–3 (0: negative, 1: weak, 2: moderate, and 3: intense immunoreactivity), and proportion was scored 0–100%. When the intensity was scored 2–3, the proportion score (%) were used for final score. When the intensity was scored 0–1, final score was scored 0. The final scores were used for clustering analysis. Evaluation was made by double blinded independent observers who were unaware of clinical data and histologic diagnoses.

### Statistical analysis

Comparative analyses of the clinicopathologic features of gastric cancer and HER2 expression between with and without p-Smad2 and c-Met were performed using the chi-squared test or Fisher’s exact test. The survival durations were calculated using the Kaplan–Meier method and analyzed by the log-rank test to compare the cumulative survival durations in the patient group. JMP 13 software (SAS Institute Japan, Tokyo, Japan) was used for the analyses.

## Results

### Clustering analysis of primary tumor in silico TCGA data and immunohistochemical staining data

**Fig. 1:**
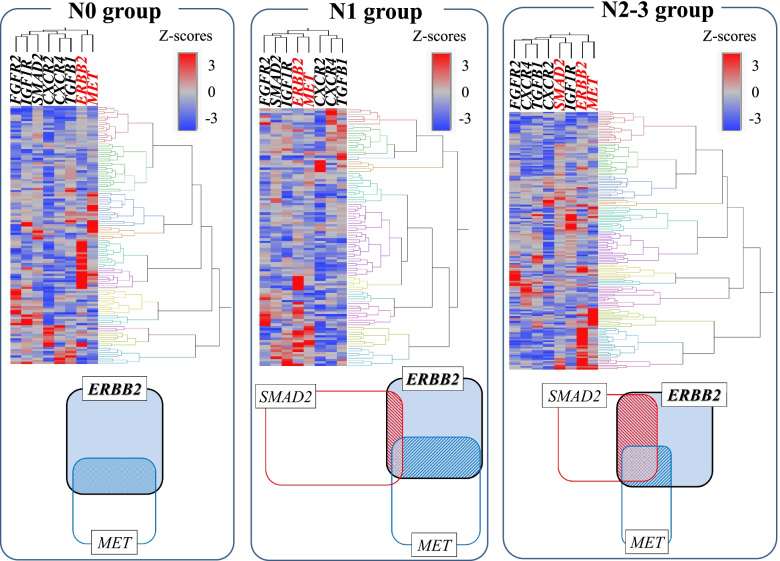
Clustering analysis of primary tumors, by using the TCGA data with each degree of lymph node metastasis. **A** In the N0 group, the *MET* amplification cases formed a cluster within the cluster with *ERBB2* amplification. **B** In the N1 group, the *MET* amplification cases formed a cluster within the cluster showing *ERBB2* amplification. The expression level in the cluster showing *ERBB2* amplification was higher than that in the N0 cases. **C** The *SMAD2* amplification cases formed a cluster within the cluster showing *ERBB2* amplification, as did the *MET* amplification cases

We performed a clustering analysis of the primary tumors by using in silico data from The Cancer Genome Atlas (TCGA) and immunohistochemical staining data based on the degree of lymph node metastasis. We divided the TCGA data regarding gastric cancer into clusters (Fig. 1). The gastric cancer group with N0 or N1 metastasis formed clusters with high expressions of *ERBB2* mRNA and *MET* mRNA. In the cluster of *MET* and *ERBB2* co-expression, the average Z-scores of *MET* m-RNA expression were 1.24 in the N0 group and 1.92 in the N1 group. In the distant lymph-node metastatic group of N2 and N3 metastasis, *SMAD2* expression was frequently observed in addition to high *ERBB2* and high *MET* expression (the Z-score of *SMAD2* m-RNA expression was 1.98 in the cluster of *MET, ERBB2*, and *SMAD2* co-expression).

**Fig. 2:**
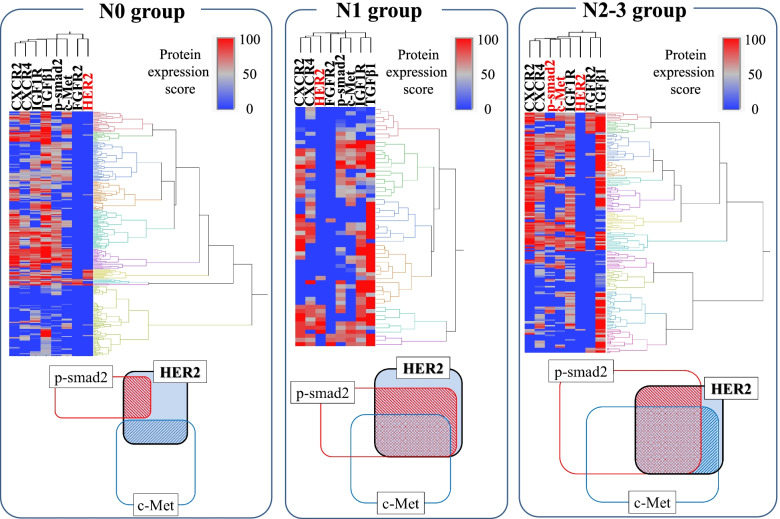
Clustering analysis of primary tumors based on the protein expression with each degree of lymph node metastasis. **A** In the clusters with high HER2 expression, the expression levels of c-Met and p-Smad2 were not very high. **B** In the clusters with high HER2 expression, the expressions of c-Met and p-Smad2 were high. **C** In the clusters with high HER2 expression, the c-Met and p-Smad2 expressions were high

We also divided the immunohistochemical staining data of the primary tumors into clusters based on the protein expression (Fig. 2). In the N0 group, the c-Met expression and p-Smad2 expression were relatively low in the HER2-overexpression cluster (the average protein expression scores of c-Met and p-Smad2 were 23.1 and 43.1, respectively). In contrast, in the N2/N3 group, high c-Met expression and high p-Smad2 expression were frequently identified in the HER2-overexpression cluster (the average protein expression scores of c-Met and p-Smad2 were 54.3 and 51.1, respectively), and this finding is consistent with the results of the in silico analysis of TCGA data.

### HER2, p-smad2, and c-Met expression in gastric cancer

**Fig. 3:**
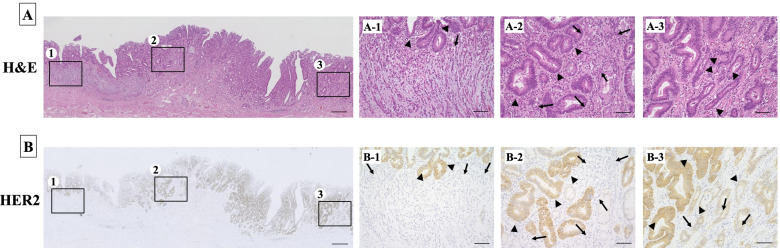
A representative gastric cancer case with intra-tumoral heterogeneity. **A** Hematoxylin and eosin (H&E) staining. Histologic intra-tumoral heterogeneity is shown. *Arrows:* poorly-differentiated cancer cells, *arrowheads:* well-differentiated cancer cells. **B** Intra-tumoral heterogeneity of HER2. *Arrowheads:* HER2-positive, *arrows:* HER2-negative. In this case, the HER2 expression was dependent on the differentiation level of the gastric cancer. The well-differentiated gastric cancer cells showed HER2-positive expression, while the poorly-differentiated cancer cells were HER2-negative (A-1, A-2, B-1, B-2). The well-differentiated cancer cells showed different HER2-positive expression levels (A-3, B-3)

**Fig. 4:**
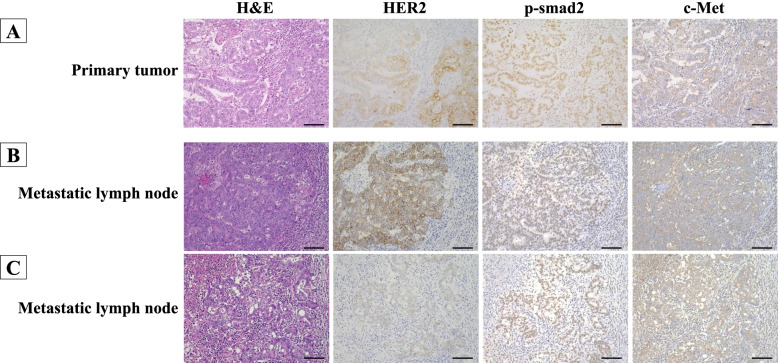
HER2-positive gastric cancer with lymph node metastasis. **A** In the primary tumor, the HER2 expression presented intra-tumoral heterogeneity. The p-Smad2 expression and c-Met expression were both high regardless of the HER2 expression. **B, C** In this metastatic lymph node, HER2 expression presented intra-tumoral heterogeneity that was similar to that of the primary tumor. The expressions of both p-Smad2 and c-Met were high regardless of the HER2 expression, as in the primary tumor

Figure 3 provides representative intra-tumoral heterogeneity of HER2 expression in a primary gastric tumor. Well-differentiated gastric cancer cells showed HER2 overexpression, whereas poorly-differentiated cancer cells were HER2-negative. Differing HER2 expression levels were also observed among the well-differentiated cancer cells. Figure 4 shows a HER2-positive gastric tumor with lymph node metastasis. HER2 heterogeneity was detected in the primary tumor, and p-Smad2 and c-Met were expressed in both HER2-negative and HER2-positive areas. In metastatic lymph nodes, the presence of both p-Smad2 expression and c-Met expression revealed heterogeneity of HER2 expression, as in the primary tumor (Fig. 4B and C).

### Relationship between the clinicopathologic features of gastric cancer and HER2 expression with or without p-Smad2 and c-Met

The details of the relationships among HER2 expression, p-Smad2 expression, and c-Met expression are provided in Table 1. A total of 43 of the 504 cases of primary GC (8.53%) were HER2-positive. In the HER2-positive gastric cancer cases, the co-expression of p-Smad2/c-Met was significantly correlated with the pathological stage (*p* = 0.042) and tended to be correlated with the T stage (*p* = 0.104) and N stage (*p* = 0.098). In the cases of HER2-negative gastric cancer, there was a significant correlation between the co-expression of p-Smad2 and c-Met and the microscopic type (*p* = 0.006).

**Table 1:** Association between clinicopathologic factors of 504 gastric cancers and p-Smad2 and/or c-Met expression in accordance to HER2 expression

**Clinico-pathologic features**	HER2-positive cases (*n* = 43)		*p*-value	HER2-negative cases (*n* = 461)		*p*-value
	Both p-smad2 and c-Met positive (*n* = 13)	Either p-smad2 or c-Met negative (*n* = 30)		Both p-smad2 and c-Met positive (*n* = 79)	Either p-smad2 or c-Met negative (*n* = 382)	
**Age**						
< 60	4 (33.3%)	8 (66.7%)		22 (16.1%)	115 (83.9%)	
≧60	9 (29.0%)	22 (71.0%)	1.000	57 (17.6%)	267 (82.4%)	0.690
**Macroscopic type**						
Bormann type 4	1 (50%)	1 (50%)		7 (13.2%)	46 (86.8%)	
Other types	12 (29.3%)	29 (70.7%)	0.518	72 (17.7%)	336 (82.3%)	0.420
**Microscopic type**						
Differentiated	11 (32.4%)	23 (67.6%)		48 (22.3%)	167 (77.7%)	
Well differentiated	2	7		12	54	
Moderately differentiated	8	16		33	111	
Papillary	1	0		3	2	
Undifferentiated	2 (22.2%)	7 (77.8%)	0.699	31 (12.6%)	215 (87.4%)	0.006
Poorly differentiated	1	6		22	143	
Signet-ring cell	0	1		7	62	
Mucinous	1	0		2	7	
Small cell	0	0		0	3	
**T stage**						
T1/T2	5 (20%)	20 (80%)		40 (15.7%)	215 (84.3%)	
T3/T4	8 (44.4%)	10 (55.6%)	0.104	39 (18.9%)	167 (81.1%)	0.358
**Lymph node metastasis**						
Negative	3 (15.8%)	16 (84.2%)		37 (14.5%)	218 (85.5%)	
Positive	10 (41.7%)	14 (58.3%)	0.098	32 (20.4%)	164 (79.6%)	0.096
**Metastasis**						
Negative	12 (28.6%)	30 (71.4%)		78 (17.4%)	371 (82.6%)	
Positive	1 (100%)	0 (0%)	0.302	1 (8.3%)	11 (91.7%)	0.700
**Pathological stage**						
I/II	5 (18.5%)	22 (81.5%)		50 (17.0%)	245 (83.0%)	
III/IV	8 (50%)	8 (50%)	0.042	29 (17.5%)	137 (82.5%)	0.887

### Survival

**Fig. 5:**
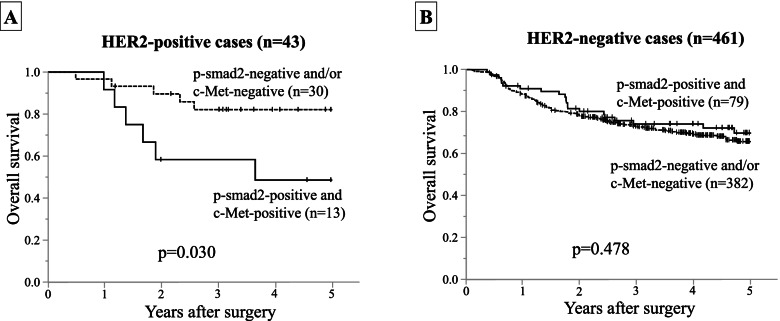
Survival curves. **A** The overall survival (OS) of the HER2-positive gastric cancer patients with a p-Smad2-positive and c-Met positive tumor was significantly poorer (*p* = 0.030) than that of the patients with a p-Smad2-negative and/or c-Met-negative tumor. **B** There was no significant difference (*p* = 0.478) in the OS of the HER2-negative gastric cancer patients with a p-Smad2-positive and c-Met positive tumor and that of the patients with a p-Smad2-negative and/or c-Met-negative tumor

In the HER2-positive gastric cancers, p-Smad2 and c-Met expression was associated with a poorer outcome. The overall survival (OS) of the HER2-positive gastric cancer patients with p-Smad2 and c-Met expression (*n* = 13) was significantly poorer than that of the patients with p-Smad2-negative and/or c-Met-negative expression (*n* = 30) (*p* = 0.030) (Fig. 5A). In contrast, there was no significant difference in the OS of the HER2-negative gastric cancer patients with both p-Smad2 and c-Met expression (*n* = 79) and the patients with p-Smad2-negative and/or c-Met-negative expression (*n* = 382) (*p* = 0.478) (Fig. 5B).

## Discussion

HER2 overexpression is found in about 15% of gastric cancers, and HER2 is the only predictive biomarker of patient’s responses to targeted therapy in gastric cancer. Our present findings related that the HER2 expression pattern in the primary tumor was dependent on the differentiation of the cancer cells. Most of the well-differentiated cancer cells showed HER2-positive expression, and most of the poorly-differentiated cancer cells showed HER2-negative expression, which is similar to previous findings [19–21]. In the present cases of HER2-positive GC, the prognosis of the patients with both p-Smad2-positive and c-Met-positive gastric cancer was significantly poorer than the prognosis of other cases. The results of the cluster analysis also indicated that in HER2-positive gastric cancer, the co-expression of p-Smad2 and c-Met was related to lymph node metastasis. These findings suggest that HER2-positive gastric tumors co-expressing p-Smad2 and c-Met might have higher malignant potential than those with either negative marker’s expression.

Not only inter-patient heterogeneity of HER2-positive cases but also inter-tumoral heterogeneity of HER2 was frequently observed in the HER2-positive tumors. In the HER2-positive gastric tumors, the expression status of both p-Smad2 and c-Met was positive in both the primary tumor and metastatic lymph nodes, and the p-Smad2 and c-Met expression levels in the metastatic lymph nodes tended to be higher than those in the primary tumors. These results suggest that the expression of c-Met and that of p-Smad2 might be correlated with lymph node metastasis in HER2-positive gastric cancer. The heterogeneity may provide the opportunity for clonal evolution, and the intra-tumoral heterogeneous clonal evolution of signaling such as that by p-Smad2 and c-Met might be associated with the acquisition of lymph-node metastatic ability in gastric cancer cells. These findings may contribute to the development of new molecular target therapies against lymph-node metastasis in patients with HER2-positive gastric cancer.

It has been reported that c-Met is frequently overexpressed in HER2-positive breast cancer cells and that c-Met contributes to trastuzumab resistance in HER2-positive breast cancer [22]. The overexpression of c-Met was observed in HER2-positive gastric cancer patients [23, 24]. *MET* amplification has been proposed as a resistance mechanism against HER2 therapy in gastric cancer [25, 26]. This resistance mechanism may be caused by the activation of MET receptor tyrosine kinase (RTK), which restores downstream signaling pathways such as those for MAPK and AKT [25]. These findings suggest that a combination of a HER2-inhibitor and a c-Met-inhibitor might exert a greater effect than a HER2-inhibitor alone in HER2-positive gastric cancer patients with lymph node metastasis.

Smad-2 is phosphorylated via TGFβ1/TGFβ1 receptor signaling, which acts in tumor progression [14, 27, 28]. It has been reported that HER2 and the TGFβ1-SMAD pathway are correlated and that there are synergistic effects of TGFβ and HER2 in the progression of breast cancer [29, 30]. It was also observed that HER2 activated the tumor-progression effect by TGFβ1 [29, 31]. Several mechanisms by which HER2 regulates TGFβ signaling have been described. ERK, p38 MAPK and PI3K/AKT have been suggested to mediate the TGFβ–induced migration and invasion in HER2-positive breast cancer [32]. These findings suggest that the co-expression of HER2 and p-Smad2 signaling might promote tumor progression and lead to distant lymph node metastasis at the N2 and N3 stages.

In our cluster analysis of the TCGA data of gastric cancer patients with N2 and N3 metastasis, the cases with high *ERBB2* expression formed clusters with cases that showed high *SMAD2* expression, but not with the cases with a high expression of *TGFB1*, which is an upstream cytokine of *SMAD2* signaling. We evaluated the expression of TGFβ1 in cancer cells alone in the immunohistochemical staining, and the TCGA data included the information from both cancer cells and tumor stromal cells. One of the reasons for the discrepancy between the TCGA data and the immunohistochemical data regarding *SMAD2* expression and TGFβ1 expression might be due to the difference in the cells evaluated in the TCGA and immunohistochemical data.

This study has some limitations. This study is only retrospective study, especially analysis with protein expression was conducted in a single department. Prospective randomized studies of gastric cancer might be necessary to clarify our result and we would like to conduct in future study.

In conclusion, intra-tumoral heterogeneity of HER2 was frequently present in HER2-positive gastric cancer. p-Smad2 and c-Met signaling might play important roles in lymph node metastasis in HER2-positive gastric cancer. The use of a c-Met inhibitor or a p-Smad2 inhibitor in combination with a HER2 inhibitor might be promising against lymph node metastasis in patients with HER2-positive gastric cancer. The expressions of p-Smad2 and c-Met might be predictive markers for prognosis and for targeting therapy in patients with HER2-positive gastric cancer.

## Supplementary Information

**Additional file 1: Figure S1.** Survival curves of TCGA patients in accordance to ERBB2.

**Additional file 2: Figure S2.** A representative figure of HER2 expression.

## Data Availability

The data supporting the study’s findings are not publicly available because they contain potentially sensitive information. However, the data can be obtained from the corresponding author or the Ethics Committee (ethics@med.osaka-cu.ac.jp) on reasonable request.

## Abbreviations

GC: Gastric cancer
TCGA: The Cancer Genome Atlas
FGFR2: Fibroblast growth factor receptor 2
TGFβ1: Transforming growth factor-beta 1
CXCR: C-X-C chemokine receptor
