# Supplementary material for: Significance of tumor heterogeneity of p-Smad2 and c-Met in HER2-positive gastric carcinoma with lymph node metastasis
Source: BMC Cancer. 2022 Jun 1;22:598. doi: 10.1186/s12885-022-09681-3 (PMC9161565; doi:10.1186/s12885-022-09681-3)
Supplement: Supplementary file 1 — Additional file 1: Figure S1. Survival curves of TCGA patients in accordance to ERBB2. [file 12885_2022_9681_MOESM1_ESM.pptx]

## Slide 1
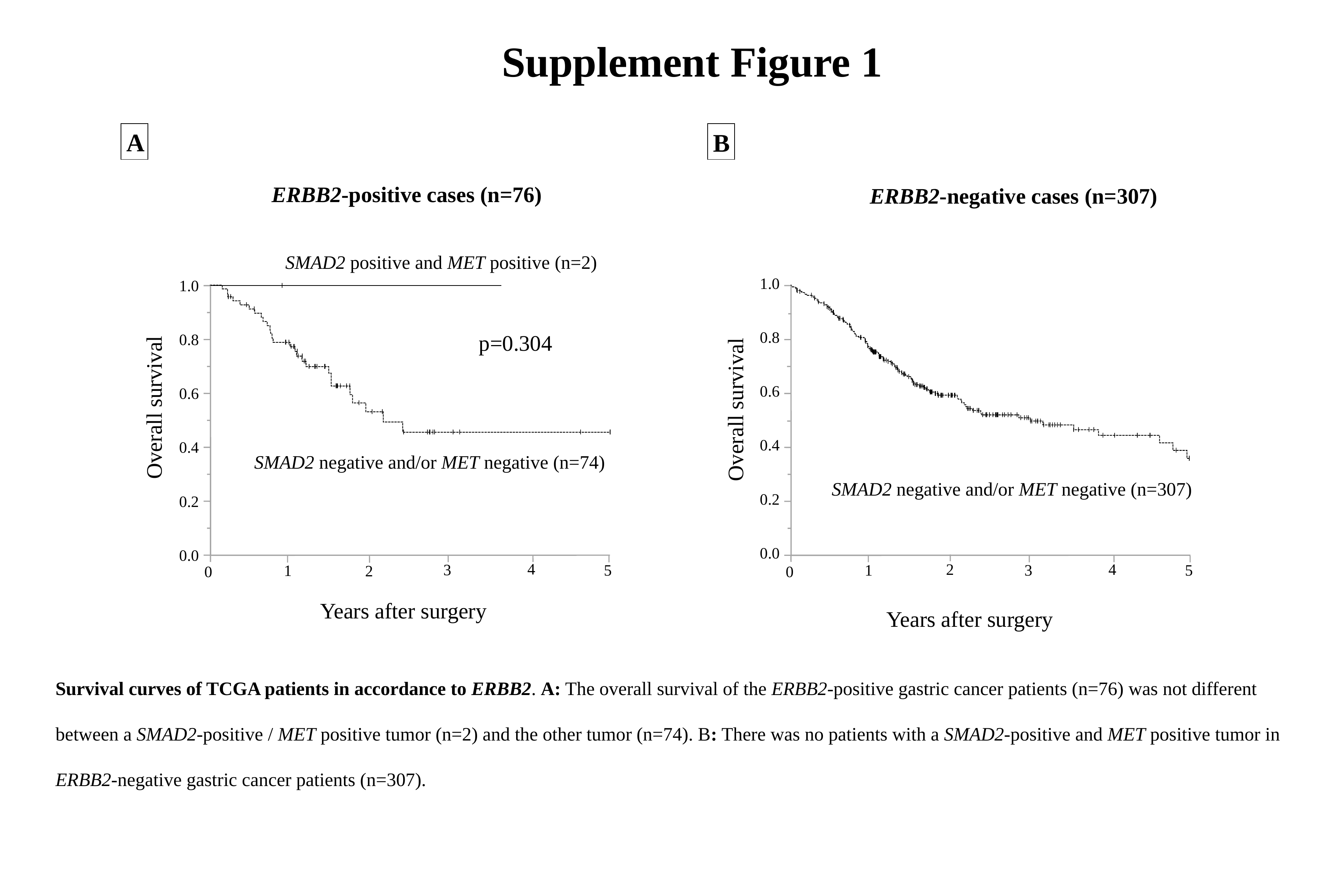

Supplement Figure 1
A
B
ERBB2-positive cases (n=76)
SMAD2 positive and MET positive (n=2)
1.0
p=0.304
0.8
0.6
Overall survival
0.4
SMAD2 negative and/or MET negative (n=74)
0.2
0.0
4
3
5
1
2
0
Years after surgery
ERBB2-negative cases (n=307)
1.0
0.8
0.6
Overall survival
0.4
SMAD2 negative and/or MET negative (n=307)
0.2
0.0
2
4
3
1
5
0
Years after surgery
Survival curves of TCGA patients in accordance to ERBB2. A: The overall survival of the ERBB2-positive gastric cancer patients (n=76) was not different between a SMAD2-positive / MET positive tumor (n=2) and the other tumor (n=74). B: There was no patients with a SMAD2-positive and MET positive tumor in ERBB2-negative gastric cancer patients (n=307).
