# Supplementary material for: Significance of tumor heterogeneity of p-Smad2 and c-Met in HER2-positive gastric carcinoma with lymph node metastasis
Source: BMC Cancer. 2022 Jun 1;22:598. doi: 10.1186/s12885-022-09681-3 (PMC9161565; doi:10.1186/s12885-022-09681-3)
Supplement: Supplementary file 2 — Additional file 2: Figure S2. A representative figure of HER2 expression. [file 12885_2022_9681_MOESM2_ESM.pptx]

## Slide 1
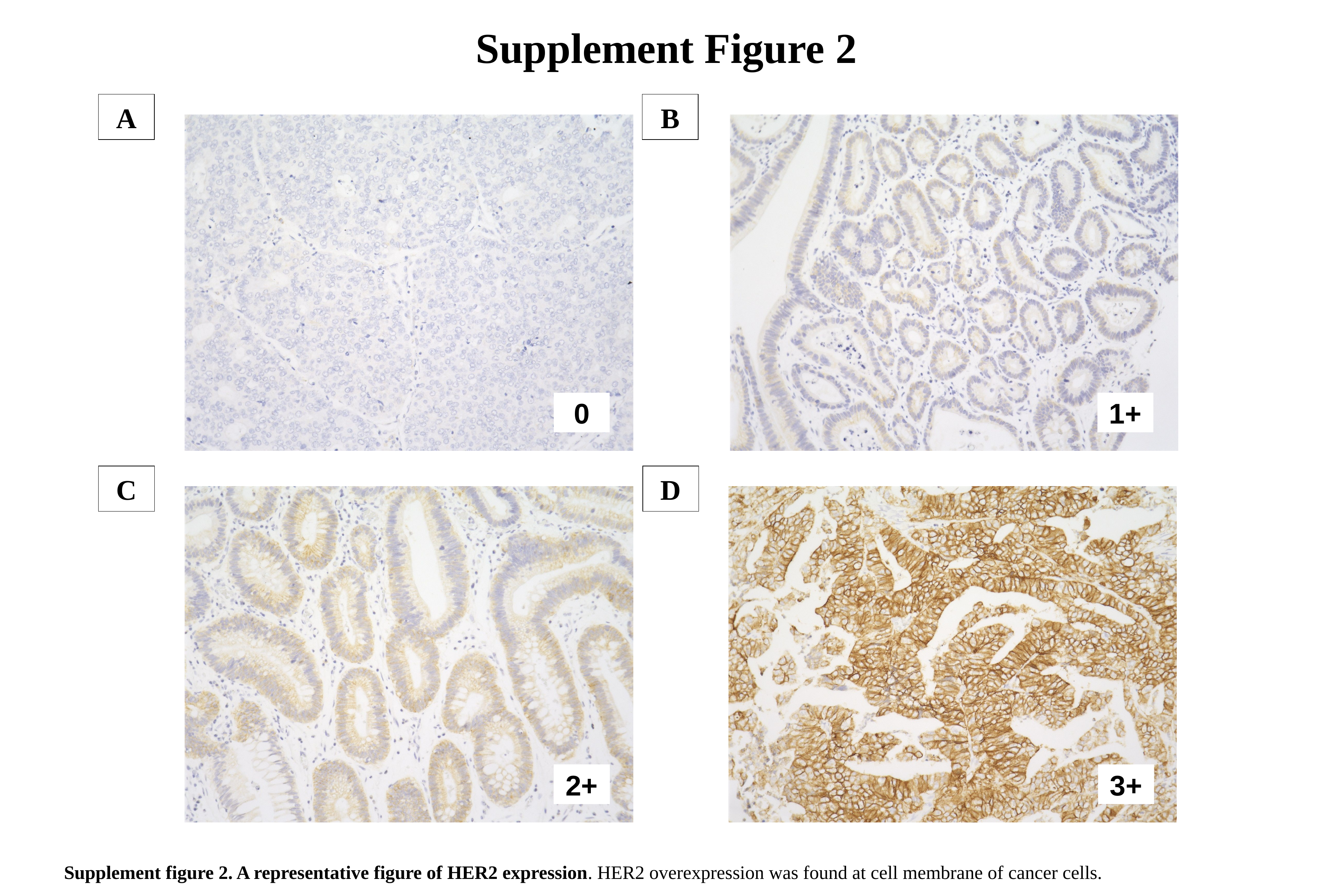

Supplement Figure 2
A
B
0
1+
C
D
2+
3+
Supplement figure 2. A representative figure of HER2 expression. HER2 overexpression was found at cell membrane of cancer cells.
